# Evidence for increased DNA damage repair in the postmortem brain of the high stress-response group of schizophrenia

**DOI:** 10.3389/fpsyt.2023.1183696

**Published:** 2023-08-22

**Authors:** Risa Shishido, Yasuto Kunii, Mizuki Hino, Ryuta Izumi, Atsuko Nagaoka, Hideki Hayashi, Akiyoshi Kakita, Hiroaki Tomita, Hirooki Yabe

**Affiliations:** ^1^Department of Neuropsychiatry, School of Medicine, Fukushima Medical University, Fukushima, Japan; ^2^Department of Disaster Psychiatry, International Research Institute of Disaster Science, Tohoku University, Sendai, Japan; ^3^Department of Pathology, Brain Research Institute, Niigata University, Niigata, Japan

**Keywords:** schizophrenia, postmortem brain, heterogeneity, environmental factor, stress, RNA-Seq, DNA double-strand break, DNA double-strand break repair by homologous recombination

## Abstract

**Background:**

Schizophrenia (SZ) is a disorder diagnosed by specific symptoms and duration and is highly heterogeneous, clinically and pathologically. Although there are an increasing number of studies on the association between genetic and environmental factors in the development of SZ, the actual distribution of the population with different levels of influence of these factors has not yet been fully elucidated. In this study, we focused on stress as an environmental factor and stratified SZ based on the expression levels of stress-responsive molecules in the postmortem prefrontal cortex.

**Methods:**

We selected the following stress-responsive molecules: interleukin (IL) -1β, IL-6, IL-10, tumor necrosis factor-α, interferon-γ, glucocorticoid receptor, brain-derived neurotrophic factor, synaptophysin, S100 calcium-binding protein B, superoxide dismutase, postsynaptic density protein 95, synuclein, apolipoprotein A1 (ApoA1), ApoA2, and solute carrier family 6 member 4. We performed RNA sequencing in the prefrontal gray matter of 25 SZ cases and 21 healthy controls and conducted a hierarchical cluster analysis of SZ based on the gene expression levels of stress-responsive molecules, which yielded two clusters. After assessing the validity of the clusters, they were designated as the high stress-response SZ group and the low stress-response SZ group, respectively. Ingenuity Pathway Analysis of differentially expressed genes (DEGs) between clusters was performed, and Terminal deoxynucleotidyl transferase-mediated dUTP nick end labeling (TUNEL) staining was conducted on four cases each in the high and low stress-response SZ groups to validate DNA damage.

**Results:**

We found higher prevalence of family history of SZ in the low stress-response SZ group (0/3 vs. 5/4, *p* = 0.04). Pathway analysis of DEGs between clusters showed the highest enrichment for DNA double-strand break repair. TUNEL staining showed a trend toward a lower percentage of TUNEL-positive cells in the high stress-response SZ group.

**Conclusion:**

Our results suggest that there are subgroups of SZ with different degrees of stress impact. Furthermore, the pathophysiology of these subgroups may be associated with DNA damage repair. These results provide new insights into the interactions and heterogeneity between genetic and environmental factors.

## 1. Introduction

Symptoms of schizophrenia (SZ), such as auditory hallucinations, delusions, and cognitive impairments, often lead to social and occupational dysfunction; thus, the unemployment rate for patients with SZ is remarkably high (80–90%) (1), resulting in enormous social and economic losses. However, the current pharmacotherapy that mainly targets dopamine D2 receptors do not sufficiently improve the symptoms of SZ (2), especially for negative symptoms and cognitive impairments that significantly impact social functioning.

SZ is generally diagnosed based on presenting symptoms, as mentioned above, for a certain period. The course and characteristics of the symptoms are heterogeneous and classically classified as disorganized, delusional, or catatonic. In addition, differences in responses to antipsychotics were also recognized as heterogeneity. To elucidate the pathogenesis of SZ, which is thought to be a collection of diseases with different pathologies, an increasing number of studies have identified subgroups within SZ. There have been multiple approaches to study heterogeneity, including classification by family history of psychiatric diseases (3, 4), differences in molecular profiles of postmortem brain (5–7), and imaging findings (8, 9). Among them, the existence of a high-inflammatory group in SZ has been repeatedly demonstrated (5, 6, 10), including a report of decreased expression of neuronal progenitor cell markers in high-inflammatory SZ (5). However, no definitive subgroup with different biological pathologies have been identified. Previous studies have shown that the development of SZ might result from the interaction between genetic and environmental factors (11), and there are likely groups within the SZ that differ in the degree of influence of these two factors, leading to heterogeneity.

The onset of SZ often occurs after adolescence, and the symptoms take time to manifest completely. Additionally, psychosocial stresses, such as failure in examinations, disappointment in love, or heavy responsibilities on the job, often trigger the onset or worsening of this disease. There have been several studies on the association of genetic factors with various environmental factors such as Toxoplasma and other infectious diseases, marijuana use, and psychosocial stress/childhood abuse at the onset of SZ. It has been reported that sensitivity to stress differed among patients with psychiatric disorders and healthy individuals depending on genetic polymorphisms in catechol-*O*-methyltransferase, an enzyme that metabolizes catecholamines, including dopamine, but the results were inconsistent among studies (12–15). Brain-derived neurotrophic factor (BDNF) (12), calcium voltage-gated channel subunit alpha1C (13), and FK506 binding protein 5 (16) have been reported as candidate genes involved in the genetic association of psychosocial stress and adverse childhood experiences with psychotic symptoms. Thus, the vulnerability-stress model, which proposes that the interaction between genetic vulnerability and psychosocial stress contribute to the pathogenesis of SZ, has been widely accepted.

While it remains unclear how stress contributes to the pathogenesis of SZ, findings regarding the participation of inflammatory responses are increasing. A study showed that the inflammation-modulating effects of cortisol, secreted through the hypothalamic–pituitary–adrenal axis, were altered in SZ (17). Further studies showed that childhood abuse increased multiple blood inflammatory markers in both humans and mice, including C-reactive protein (18), interleukin (IL) -1β, IL-6, and tumor necrosis factor (TNF) -α (19). Thus, it is becoming clear that stress can trigger abnormal immune system activity in SZ, and there is a trend to identify biomarkers from the immune system (20), although it has been pointed out that increased inflammation is not common to all types of SZ (21).

Although many studies have explored the interaction of genetic and environmental factors with SZ, it remains to be understood whether there are subgroups that differ in the strength of the influence of each factor. To clarify how genetic and environmental factors contribute to the pathogenesis of SZ, it is necessary to identify subgroups that differ in their degree of influence and examine the pathogenesis of each subgroup. This would reduce the influence of heterogeneity and bring us closer to the essence of the pathological basis of SZ. Detailed biological studies using postmortem brains are needed to clarify the existence of subgroups with different degrees of influence of genetic and environmental factors on SZ and the pathophysiological differences between them. However, only a few of these studies have been conducted. In the present study, we focused on stress as an environmental factor and conducted a cluster analysis of SZ based on the expression levels of stress-responsive molecules in the prefrontal cortex. We aimed to (i) identify a subgroup of people with SZ who display increased stress response, (ii) investigate differences in pathophysiology between subgroups using clinical information and pathway analysis of differentially expressed genes (DEGs), and (iii) validate these differences using histochemical methods. We hypothesized that the high stress-response SZ group may have a reduced genetic vulnerability to SZ compared to the lower stress-response SZ group, and these groups may have different mechanisms of SZ pathogenesis.

## 2. Subjects and methods

### 2.1. Human postmortem brain tissue collection and characterization

Postmortem brain tissue samples from patients with SZ and control subjects were obtained from the Fukushima Brain Bank at the Department of Neuropsychiatry, School of Medicine, Fukushima Medical University (Fukushima, Japan), and the Brain Research Institute at Niigata University (Niigata, Japan), as described previously (22). The use of postmortem human brain tissue was approved by the Ethics Committee of Fukushima Medical University and Niigata University and complied with the Declaration of Helsinki and its later amendments. Patients with SZ fulfilled the diagnostic criteria established by the American Psychiatric Association (Diagnostic and Statistical Manual of Mental Disorders, DSM-IV). Control refers to individuals who had not been diagnosed with a mental illness, according to the DSM-IV, during their lifetime. The Diagnostic Instrument for Brain Studies (DIBS) was used to evaluate the antemortem symptoms of each patient with SZ 3 months before their death (23). We classified each item of the antemortem symptoms of DIBS into three subscales: positive symptoms, negative symptoms, and general psychopathology, according to the Positive and Negative Syndrome Scale (24). The highest score for each item was used for their lives. For the gene expression studies, we used mRNA from the gray matter of the prefrontal cortex (PFC) corresponding to Brodmann area 10 of 25 patients with SZ and 21 control subjects. This is because the PFC is considered to be closely related to negative symptoms and cognitive dysfunction, and atrophy of spines and dendrites of PFC neurons and altered gene expression have been reported (25). The detailed demographic information for each group is summarized in Table 1.

**Table 1:** Demographic data of samples for RNA-seq.

	Control	Schizophrenia	*p*-value
Number of samples	21	25	
Age of death (±SD)	66.4 ± 17.7	68.4 ± 11.3	0.66^a^
Sex	8F, 13 M	8F, 17 M	0.67^b^
RIN (±SD)	5.8 ± 1.2	7.4 ± 0.9	<0.001^a^
PMI (±SD) (h)	11.4 ± 12.9	16.8 ± 11.8	0.16^a^
Illness duration (±SD) (year)	-	41.0 ± 9.4	
CPZ-eq (±SD)	-	559.1 ± 595.1	

RIN, RNA integrity number; PMI, postmortem interval; CPZ-eq, chlorpromazine equivalent of total antipsychotics; ^a^Student’s t-test, ^b^*χ*^2^-test

### 2.2. RNA isolation, sequencing, mapping, and normalization

RNA isolation and mRNA sequencing were performed at 2 × 100 bp using HiSeq4000 (Illumina, Tokyo, Japan), yielding an average of 46.6 million reads per individual, as described previously (26). The reads were cleaned using Trimmomatic (ver. 0.39)^1^ and mapped to the Ensembl human genome GRCh38.p13 as a reference using BWA (ver. 0.7.17).

### 2.3. Stress-responsive molecules

Based on the previous findings, the glucocorticoid receptors (GR), inflammatory cytokines (27), and redox regulatory mechanisms (28) have been assumed to be associated with stress-induced molecular mechanisms. However, there are many aspects of stress-induced molecular mechanisms that remain unclear. Therefore, in this study, we considered “stress-responsive molecules” as those whose expression were reported to be altered under stress by multiple studies and many of these are involved in the signaling mechanisms of molecular pathways described above.

The following molecules were selected as stress-responsive molecules: (i) molecules reported to be upregulated by stress, namely IL-1β, IL-6, TNF-α (19, 29), interferon (IFN)-γ (19, 30), and superoxide dismutase (SOD)1, SOD2, SOD3 (31), (ii) molecules reported to be downregulated by stress, namely IL-10 (29), BDNF, postsynaptic density protein (PSD) 95, synaptophysin (SYP) (32, 33), α-synuclein (SNCA), β-synuclein (SNCB), and γ-synuclein (SNCG) (34, 35), and (iii) molecules whose expression reported to be altered by stress, however, the direction of change in expression by stress varies from literature and is inconsistent, namely apolipoprotein A1 (ApoA1) and ApoA2 (36, 37), GR (38), S100 calcium binding protein B (S100B) (39, 40) and solute carrier family 6 member 4 (SLC6A4) (41). The stress-responsive molecules used are listed in Table 2.

**Table 2:** Stress-responsive molecules.

	Protein (gene)	Major role	Type of stress
Increase with stress	IL-1β (*IL1B*)	Inflammation, immune system	Infection, psychosocial stress (28, 35)
	IL-6 (*IL6*)		
	IFN-γ (*IFNG*)		Psychosocial stress (19)
	TNF-α (*TNF*)		
	SOD-1, −2, −3 (*SOD1,2,3*)	Antioxidant enzyme	Oxidative stress (36), chronic stress (31)
Decrease with stress	BDNF (*BDNF*)	Neurotrophic factor	Psychosocial stress (32), chronic stress (33)
	IL-10 (*IL10*)	Inflammation, immune system	Psychosocial stress (29)
	PSD95 (*DLG4*)	Synaptogenesis, synaptic plasticity	Psychosocial stress (32), chronic stress (33)
	Synaptophysin (*SYP*)	Synaptic plasticity	
	Synuclein-α (*SNCA*)	Integrate presynaptic signaling and membrane trafficking	Oxidative stress (34, 36), psychosocial stress (35)
	Synuclein-β (*SNCB*)	Act as a regulator of SNCA aggregation process	
	Synuclein-γ (*SNCG*)	Neurofilament network integrity	
Inconsistent studies of stress-induced expression	ApoA1, 2 (*APOA1, 2*)	Cholesterol transport	Oxidative stress (36)
	GR (*NR3C1*)	Receptor of cortisol	Psychosocial stress (6)
	S100B (*S100B*)	Neurite extension, proliferation of melanoma cells, stimulation of Ca2+ fluxes, astrocytosis and axonal proliferation, and inhibition of microtubule assembly	Oxidative stress (40)
	5HTT (*SLC6A4*)	Terminates the action of serotonin and recycles it in a sodium-dependent manner.	Maternal stress, child abuse, psychosocial stress (41)

IL, interleukin; IFN, interferon; TNF, tumor necrosis factor; SOD, superoxide dismutase; BDNF, brain-derived neurotrophic factor; PSD, postsynaptic density protein; SYP, synaptophysin; HDL, high-density lipoprotein; Apo, apolipoprotein; GR, glucocorticoid receptor; S100B, S100B calcium-binding protein B; SLC6A4, solute carrier family 6 member 4.

### 2.4. Cluster analysis of SZ cases by mRNA expression levels of stress-responsive molecules

Cluster analysis was performed using the Ward method to clusterise SZ cases by mRNA of stress-responsive molecules. In this study, the mRNA expression levels of each stress-responsive molecule were standardized using Z-scores.

### 2.5. Analysis of DEGs, pathway analysis

We used the edgeR package in R (version 4.1.2) to calculate the *value of p* of the Student’s *t*-test and log_2_ fold changes in mRNA expression levels of each gene between groups. The *value of ps* and log_2_ fold-change values of 8,000 genes selected in order of increasing *value of ps* were input into the Ingenuity Pathway Analysis for canonical pathway analysis.

### 2.6. Visualization of 3’-OH termini of DNA in paraffin tissue section

Formalin-fixed paraffin-embedded tissue sections (7 μm thick) from Brodmann area 10 of eight SZ patients (four patients of each high and low stress-response SZ group) (Table 3) were processed with proteinase K (20 μg/mL) (EMD Millipore, Temecula, CA, United States) directly on the slides (60 μL/ cm^2^) for 15 min at room temperature (25°C), followed by Terminal deoxynucleotidyl transferase-mediated dUTP nick end labeling (TUNEL) staining by Apop Tag Plus Peroxidase *in situ* apoptosis detection kit (EMD Millipore, Temecula, CA, United States) as manufacturer’s instruction. The specimens were stained with methyl green (Muto Pure Chemicals, Tokyo, Japan) and observed under a microscope (BX51, Olympus, Tokyo, Japan) with × 40 [numerical aperture (NA) = 0.75] and × 60 (NA = 1.42) objective lens. All images were captured under the same conditions using an RGB camera (α7S II, Sony, Tokyo, Japan). For the determination of the TUNEL positivity rate, six images were captured per case in the third cortical layer under a × 40 objective lens. We used an Imaging Edge Desktop (Sony) to process the images. ImageJ (42) was used to count TUNEL-positive and methyl green-positive cells. The TUNEL-positive area stained with diaminobenzidine and methyl green-positive area were extracted separately using the “Color Deconvolution” plugin, and each was binarized using the “Threshold” tool with the default settings. Then, TUNEL-positive and methyl green-positive cells were counted using the “Analyze particles” tool with a defined minimum size of 2000 pixel^2^ and circularity range of 0.1–1.

**Table 3:** Demographic data of samples for TUNEL staining.

	Age of death	Sex	RIN	PMI (h)	Illness duration (year)	CPZ-eq
High stress-response SZ group	70	M	6.4	28.1	49	550
	75	M	5.8	17.17	42	225
	87	F	8	3.45	38	0
	76	F	6.1	25	48	114
Low stress-response SZ group	64	M	8.3	4.5	47	15
	55	M	7.5	3.6	28	1,550
	60	M	7.1	26.1	42	800
	86	M	8.6	14.64	58	200

RIN, RNA integrity number; PMI, postmortem interval; CPZ-eq, chlorpromazine equivalent of total antipsychotics.

### 2.7. Classification of control subjects into high and low stress responders

The control subjects were classified into high and low stress responders as follows: First, the control subjects were divided into quartiles based on the mRNA expression levels of each gene, including *IL-6, IFN-γ*, *SOD2*, *GR*, *ApoA1*, *SLC6A4, BDNF*, *SYP*, *PSD95*, and *SNCA*. The expression profiles exhibited significant differences or trends between groups with SZ, and the stress-induced increase and decrease in these molecules were consistent with the results of the previous studies (19, 29–38). For the genes *IL-6, IFN-γ*, *SOD2*, *GR*, *ApoA1*, and *SLC6A4*, which were highly expressed in the high stress-response SZ group (HSR-SZ), subjects in the first, second, third, and fourth quartiles were scored as −3, −1, 1, and 3, respectively. In addition, for genes *BDNF*, *SYP*, *PSD95*, and *SNCA*, which were highly expressed in the low stress-response SZ group (LSR-SZ), subjects in the first, second, third, and fourth quartiles were scored as 3, 1, −1, and − 3, respectively. Each score was summed to obtain the stress-response index for each participant. The group with a stress-response index greater than 0 was defined as the high stress-response control group (HSR-Cont), and the group with a stress-response index less than 0 was defined as the low stress-response control group (LSR-Cont). IPA analysis of DEGs between the control groups was performed using the same protocol as for SZ.

### 2.8. Statistical analysis

**Figure 1:**
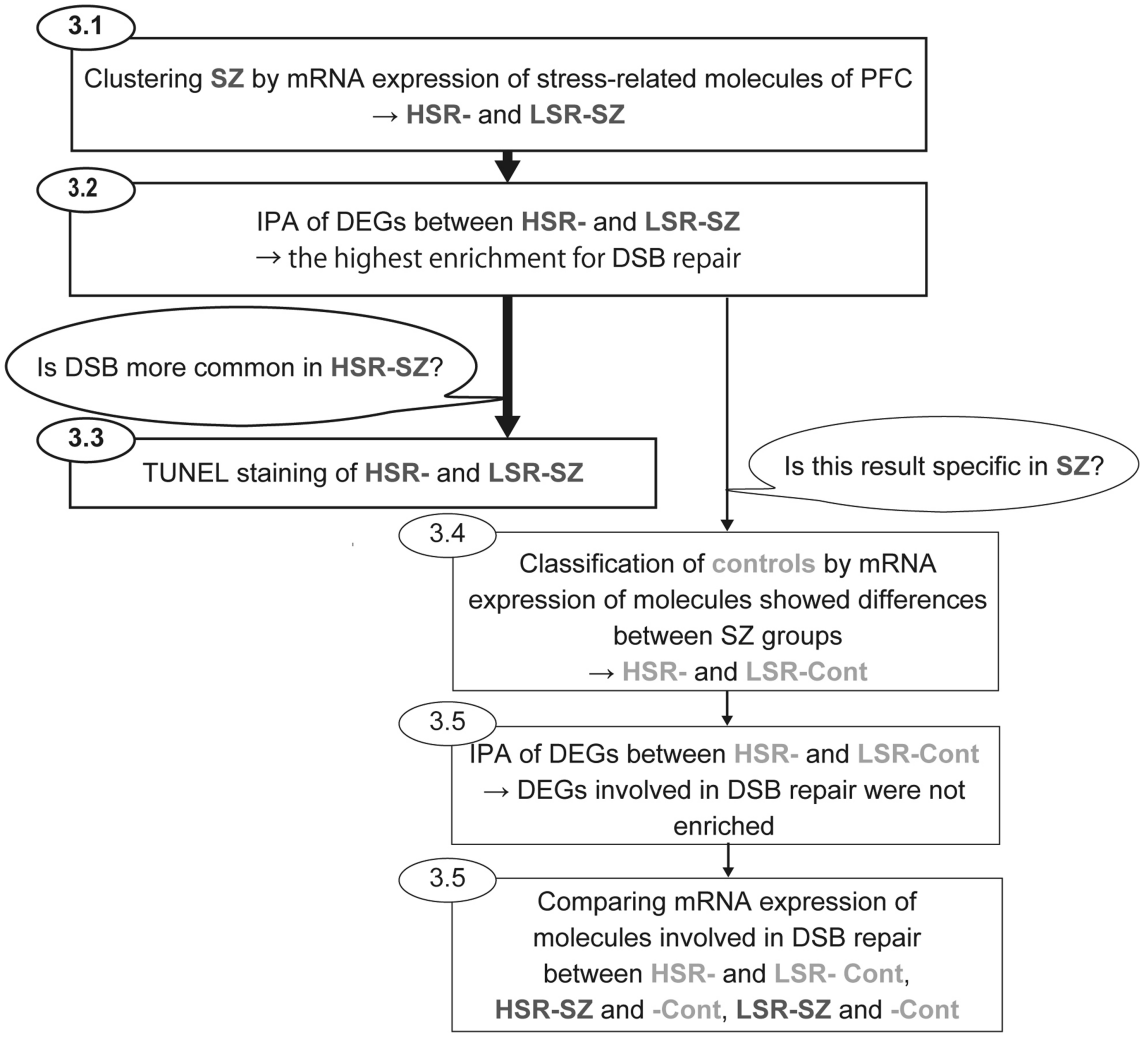
Flow of analysis for this study. First, cluster analysis of schizophrenia (SZ) by mRNA expression levels of stress-responsive molecules was performed to clusterise SZ into two groups (HSR- and LSR-SZ). Second, canonical pathway analysis of DEGs between HSR- and LSR-SZ showed the highest enrichment for DSB repair. Third, to validate whether DSB was more common in HSR-SZ than in LSR-SZ, we conducted TUNEL staining of BA10 of HSR- and LSR-SZ. In addition, to estimate whether similar results could be obtained in controls, they were classified as HSR- and LSR-Cont according to mRNA expression of stress-responsive molecules that showed differences between SZ groups. Then, we performed canonical pathway analysis of DEGs between control groups, and found no enrichment for DSB repair. Finally, mRNA expression of molecules involved in DSB repair was compared between HSR- and LSR-Cont, HSR-SZ and -Cont, LSR-SZ and -Cont. HSR-SZ, high stress-response SZ group; LSR-SZ, low stress-response SZ group; DSB, DNA double-strand break; HSR-Cont, high stress-response control group; and LSR-Cont, low stress-response control group.

Two-group comparisons of gene expression levels (HSR-SZ vs. LSR-SZ, HSR-Cont vs. LSR-Cont, HSR-SZ vs. HSR-Cont, LSR-SZ vs. LSR-Cont) and the mean TUNEL-positive cell rates (HSR-SZ vs. LSR-SZ) were performed using the Mann–Whitney *U* test. We used Fisher’s exact test to compare the distribution of categories of cause of death between HSR- vs. LSR-SZ and HSR- vs. LSR-Cont. Statistical significance was set at *p* < 0.05. SPSS ver. 28.0 (SPSS, Chicago, IL, United States) and R (version 4.1.2) were used for statistical analysis. The flow of analysis for this study is shown in Figure 1.

## 3. Results

### 3.1. Cluster analysis of SZ cases by mRNA expression of stress-responsive molecules

**Figure 2:**
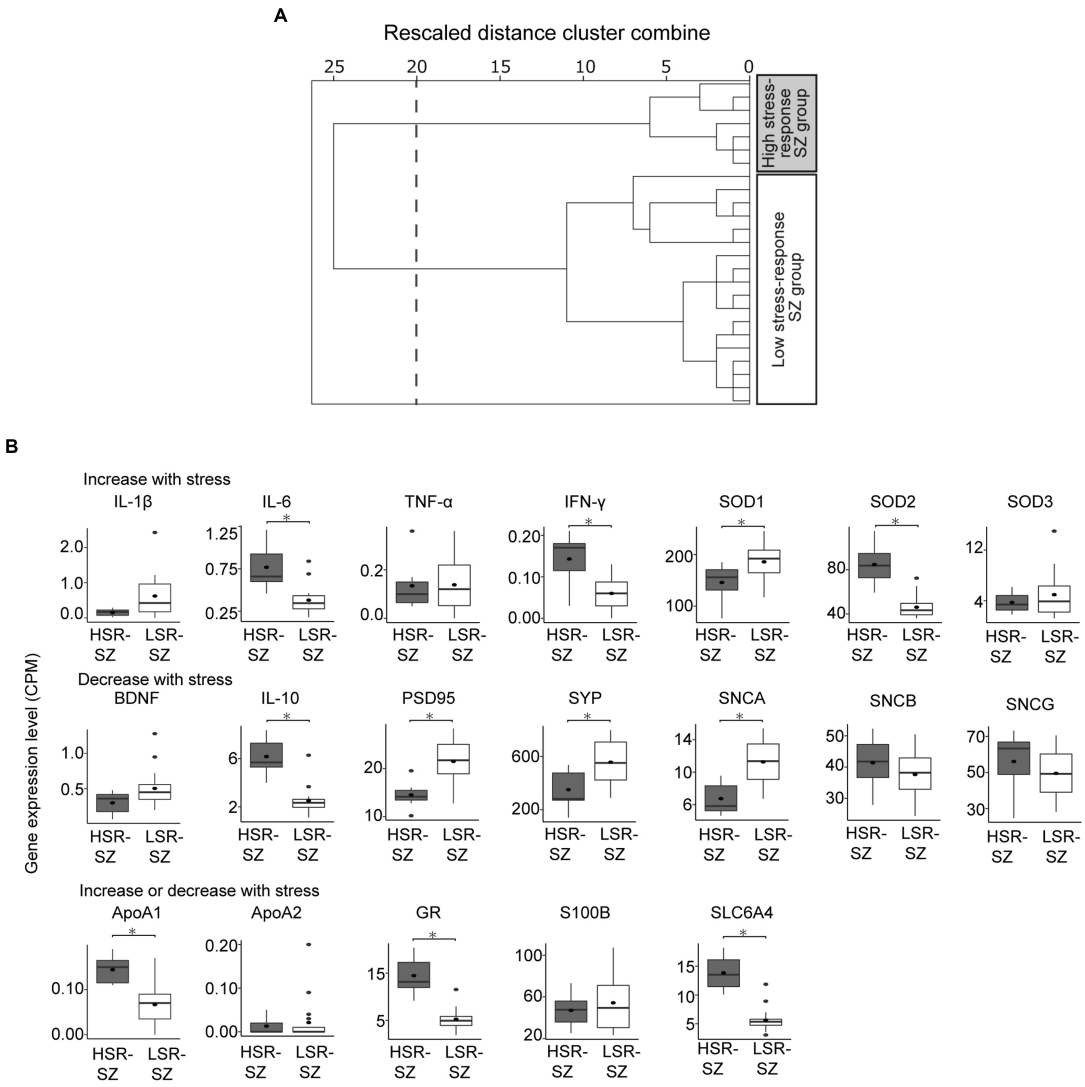
**(A)** Hierarchical clustering of patients with schizophrenia (SZ) by mRNA levels of stress-responsive molecules. Cluster analysis divided 25 patients with SZ into two clusters at the 20 rescaled distance cluster combine (dashed line). **(B)** Box-and-whisker plot of gene expression levels of stress-responsive molecules of two SZ subgroups showing the median (thick line in box), 25th and 75th percentiles (box ends), and minimum and maximum (whiskers) values. HSR-SZ, high stress-response SZ group; LSR-SZ, low stress-response SZ group; CPM, count per million; * indicates *p* < 0.05.

Hierarchical clustering by expression levels of stress-responsive molecules divided 25 SZ cases into two clusters at the 20 rescaled distance cluster combine and included 7 and 18 participants, respectively (Figure 2A). When the expression levels of stress-responsive molecules were compared between the two clusters, one cluster showed significantly higher expression of three of the molecules reported to be upregulated by stress than another cluster (IL-6 0.77 ± 0.10 vs. 0.38 ± 0.04, *p* < 0.001, IFN-γ 0.14 ± 0.02 vs. 0.06 ± 0.01, *p* = 0.006, SOD2 84.7 ± 7.22 vs. 46.1 ± 2.32, *p* < 0.001), and three of molecules reported to be lowered by stress were significantly reduced (PSD95 14.5 ± 1.0 vs. 21.5 ± 1.05, *p* = 0.002, SYP 350.1 ± 55.58 vs. 556.3 ± 38.96, *p* = 0.009, SNCA 6.74 ± 0.74 vs. 11.26 ± 0.64, *p* < 0.001), BDNF which also reported to be lowered by stress tended to be decreased (BDNF 0.30 ± 0.06 vs. 0.51 ± 0.06, *p* = 0.055). Therefore, cluster with higher expression of molecules that are elevated by stress, such as IL-6, IFN-γ, and SOD2, and lower expression of molecules that are decreased by stress, such as PSD95, SYP, SNCA, and BDNF, was defined to be the HSR-SZ. The other cluster was defined as the LSR-SZ (Figure 2B).

The HSR- and LSR-SZ did not significantly differ in PMI, age, sex, illness duration, and daily dosage of antipsychotics expressed in chlorpromazine equivalent (CPZ-eq). RIN was significantly decreased in the HSR-SZ (6.62 ± 0.7 vs. 7.75 ± 0.9, *p* = 0.01). We compared the number of relatives with SZ in patients for whom information on the family history of psychiatric disease was available (number of relatives diagnosed with SZ/patients with available information regarding the presence of family members with a psychiatric history) and found that the HSR-SZ had less family history than the LSR-SZ (0 relatives/3 patients vs. 5 relatives/4 patients, *p* = 0.04; Table 4). Since stress-responsive molecules used in this study included various inflammatory cytokines, the presence or absence of inflammation during the time of death could have influenced the result of clusterization. Hence, we categorized the causes of death in the cases into three categories: inflammatory disease, non-inflammatory disease, and malignancy, and then examined whether there were differences in the distribution of the categories between HSR- and LSR-SZ. We found no significant differences in the distribution of the categories between the two groups of SZ (*p* = 0.84; Table 5).

**Table 4:** Demographic and clinical data of high/low stress-response SZ group.

	High stress-response SZ group	Low stress-response SZ group	*p*-value
Number of samples	7	18	
Age of death (±SD)	74.3 ± 6.3	66.1 ± 12.1	0.10^a^
Sex	4F, 3 M	5F, 13 M	0.17^b^
RIN (±SD)	6.62 ± 0.7	7.75 ± 0.9	0.01^a^
PMI (±SD) (h)	18.6 ± 8.1	16.0 ± 13.1	0.64^a^
Age of onset (±SD)	31.1 ± 6.0	24.6 ± 7.9	0.07^a^
Illness duration (±SD) (year)	41.9 ± 4.8	40.7 ± 10.9	0.90^a^
CPZ-eq (±SD)	347.0 ± 345.4	651.9 ± 664.4	0.26^a^
Family history of SZ	0/3 cases	5/4 cases	0.04^b^
DIBS			
Positive symptoms	16.3 ± 6.6	13.5 ± 11.6	0.26^a^
Negative symptoms	2.3 ± 3.0	3.1 ± 2.1	0.53^a^
General psychopathology	3.6 ± 1.9	4.1 ± 3.5	1.00^a^

RIN, RNA integrity number; PMI, postmortem interval; CPZ-eq, chlorpromazine equivalent of total antipsychotics; DIBS, diagnostic instrument for brain studies; ^a^Mann-Whitney U test, ^b^*χ*^2^-test

**Table 5:** Cause of death of high/low stress-response SZ group.

	Category^a^	Cause of death
High stress-response SZ group	Inflammatory disease	Pneumonia
	Inflammatory disease	Pneumonia
	Inflammatory disease	Pneumonia
	Inflammatory disease	Pneumonia
	Non-inflammatory disease	Renal failure
	Non-inflammatory disease	Stroke
	Malignancy	Stomach cancer, Gallbladder cancer
Low stress-response SZ group	Inflammatory disease	Pneumonia
	Inflammatory disease	Pneumonia
	Inflammatory disease	Pneumonia
	Inflammatory disease	Pneumonia
	Inflammatory disease	Pneumonia
	Inflammatory disease	Pneumonia
	Inflammatory disease	Malignant syndrome
	Non-inflammatory disease	Stroke
	Non-inflammatory disease	Arrhythmia
	Non-inflammatory disease	Heart failure
	Non-inflammatory disease	Heart failure
	Non-inflammatory disease	Multiple organ failure
	Non-inflammatory disease	Die from old age
	Non-inflammatory disease	Suffocation
	Non-inflammatory disease	Suicide
	Malignancy	Lung cancer
	Malignancy	Stomach cancer
	Malignancy	Cecal cancer

a No significant difference in the distribution of categorized causes of death between the two groups of SZ by Fisher’s exact test.

### 3.2. IPA canonical pathway analysis of DEGs between HSR–and LSR-SZ

**Figure 3:**
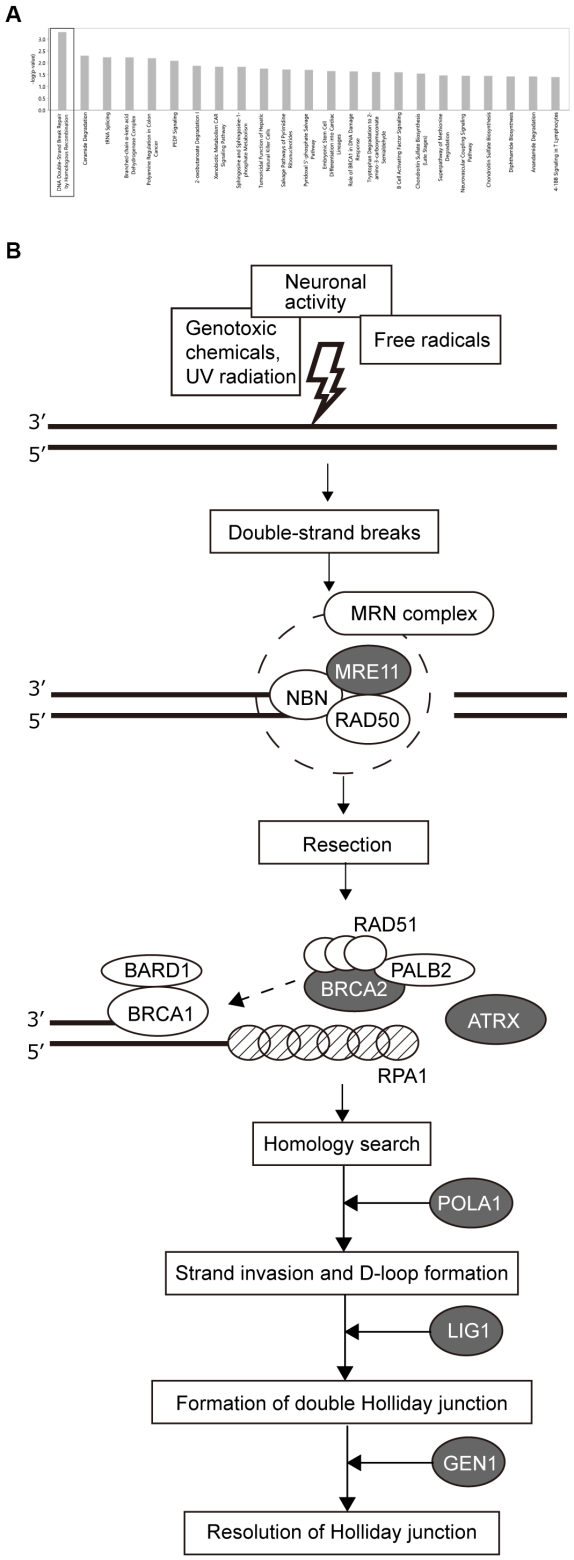
**(A)** Canonical pathway analysis of DEGs between high and low stress-response schizophrenia (SZ) groups. DEGs were most highly enriched in DSB repair by homologous recombination. **(B)** Molecules involved in the DSB repair pathway. Proteins indicated by gray ovals are those whose mRNA expression was higher in the high stress-response SZ group, and RPA1 indicated by shaded pattern ovals were where the mRNA expression was lower in the high stress-response SZ group.

To investigate the differences in pathology between the HSR- and LSR-SZ, canonical pathway analysis was performed on the DEGs (top 8,000 genes sorted by significance) between the two groups (Supplementary Table S1). Our analysis revealed the most significant enrichment of DEGs in DNA double-strand break (DSB) repair by homologous recombination (Figure 3A). Among the molecules involved in DSB repair by the homologous recombination pathway, seven molecules, namely MRE11, BRCA2, ATRX, RPA1, POLA1, LIG1, and GEN1, were included in 8000 DEGs. The mRNA expression of *MRE11*, *BRCA2*, *ATRX*, *POLA1*, *LIG1,* and *GEN1* was higher in the HSR-SZ than in the LSR-SZ, but only *RPA1* was decreased in the HSR-SZ (Figure 3B).

### 3.3. Visualization of 3’-OH termini of DNA in paraffin tissue section

**Figure 4:**
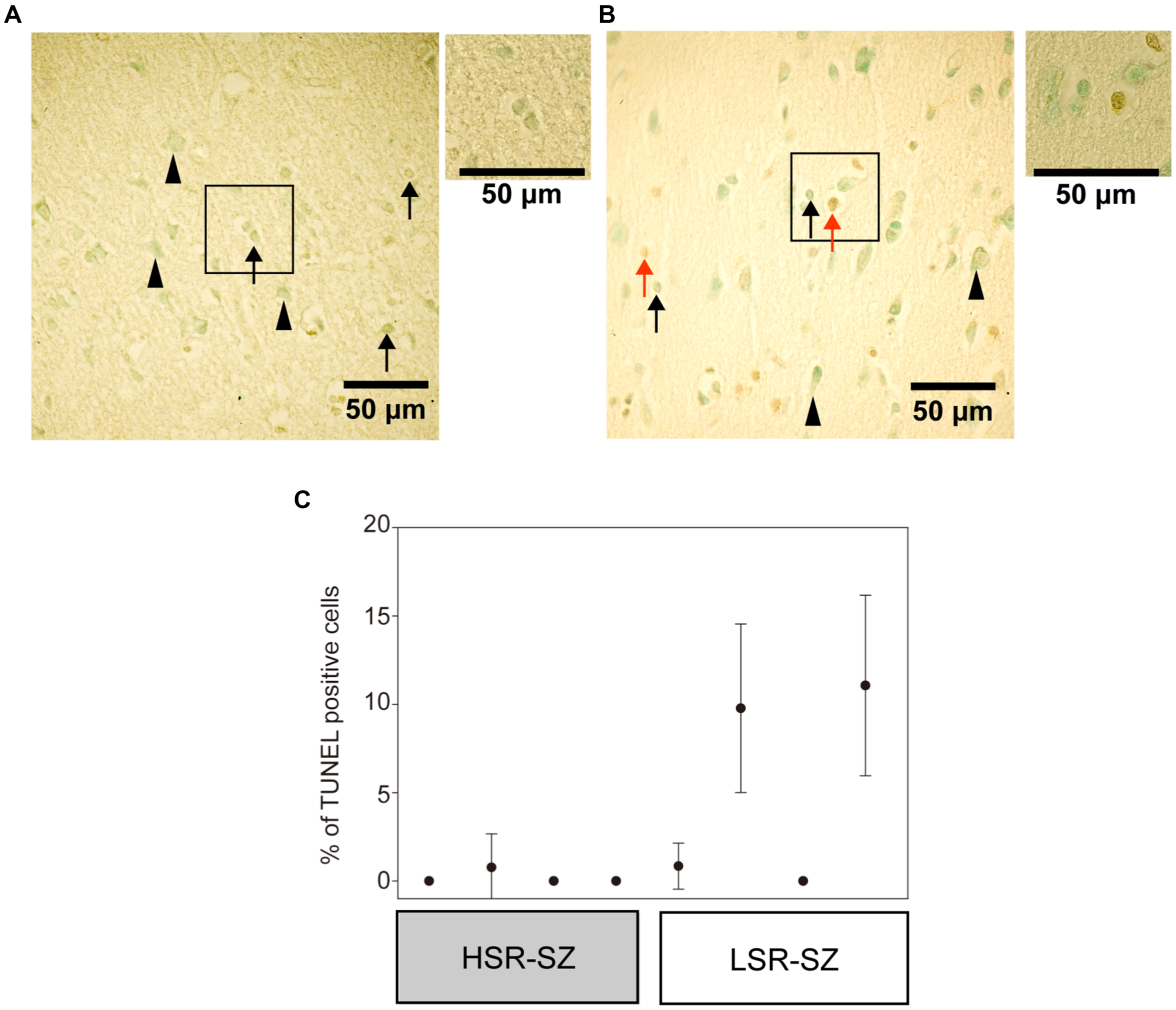
**(A,B)** Visualization of 3’-OH termini of DNA in the third layer of frontal cortex in high stress-response schizophrenia (SZ) group **(A)** and low stress-response SZ group **(B)** with a × 40 objective. The regions marked with boxes observed with a × 60 objectives were attached on the right side. Arrowheads indicate neurons, and arrows indicate astrocytes or microglia. Red arrows indicate TUNEL-positive cells **(B)**. **(C)** There were cases with a higher percentage of TUNEL-stained positive cells in the low stress-response SZ group. Error bar means standard deviation. HSR-SZ, high stress-response SZ group; LSR-SZ, low stress-response SZ group.

Next, we observed the 3’-OH termini of DNA in the tissue section of the prefrontal cortex of SZ cases with TUNEL staining (Figures 4A,B). TUNEL-positive cells were observed in glial cells (mostly astrocytes) but not in neurons. In the LSR-SZ, two samples showed remarkably high TUNEL-positive cell rates (Figure 4C). Even though the results were preliminary owing to the small sample size, the effect size of the difference in TUNEL-positive cell rates between the HSR- and LSR-SZ was large (*r* = 0.598).

### 3.4. Comparison of expression levels of stress-responsive molecules between control groups

We categorized the 21 control subjects into HSR- (*n* = 14) and LSR-Cont (*n* = 7). Comparison of mRNA expression levels of stress-responsive molecules between HSR- and LSR-Cont showed that three of the molecules (IL-6 0.59 ± 0.07 vs. 0.33 ± 0.10, *p* = 0.01, SOD2 79.6 ± 10.17 vs. 50.6 ± 4.96, *p* = 0.006, SOD3 6.95 ± 0.66 vs. 3.81 ± 0.63, *p* = 0.007) reported to be upregulated by stress were significantly higher in HSR-Cont, and three of the molecules (PSD95 15.2 ± 1.16 vs. 20.6 ± 1.74, *p* = 0.038, SYP 366.3 ± 43.4 vs. 558.5 ± 34.4, *p* = 0.004, SNCA 7.68 ± 0.81 vs. 12.12 ± 0.74, *p* = 0.002) reported to be downregulated by stress were lower in HSR-Cont (Supplementary Figure S1).

There were no significant differences in age, sex, PMI, or RIN between the two groups (Table 6). No significant differences were found between the control groups in the distribution when the causes of death were categorized as inflammatory disease, non-inflammatory disease, or malignancy (Supplementary Table S2).

**Table 6:** Demographic data of high/low stress-response control group.

	High stress-response control group	Low stress-response control group	*p*-value
Number of samples	14	7	
Age of death (±SD)	64.2 ± 20.2	70.9 ± 11.5	0.49^a^
Sex	7 M	1 F, 6 M	0.11^b^
RIN (±SD)	5.58 ± 1.0	6.23 ± 1.6	0.44^a^
PMI (±SD) (h)	13.2 ± 13.7	8.1 ± 11.4	0.54^a^

RIN, RNA integrity number; PMI, postmortem interval; ^a^Mann-Whitney U test, ^b^*χ*^2^-test

### 3.5. IPA analysis of DEGs between control groups classified by mRNA expression of stress-responsive molecules

**Figure 5:**
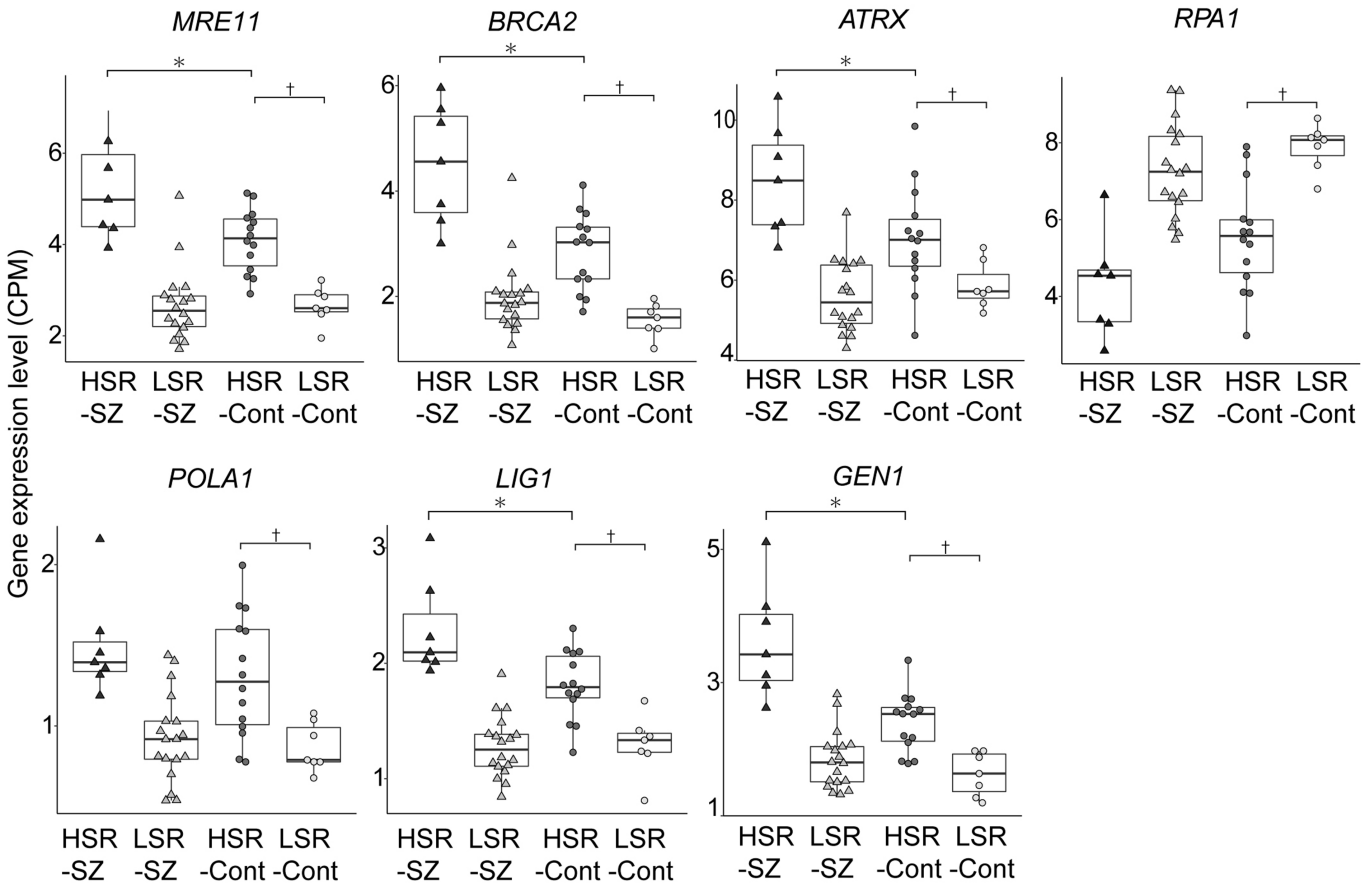
Box-and-whisker plot of gene expression levels of molecules involved in the DSB repair pathway in patients with schizophrenia (SZ) and controls. The expression levels between HSR- and LSR-Cont, HSR-SZ and -Cont, and LSR-SZ and -Cont were compared by the Mann–Whitney U test, respectively. CPM, count per million; HSR-SZ, high stress-response SZ group; LSR-SZ, low stress-response SZ group; HSR-Cont, high stress-response control group; LSR-Cont, low stress-response control group; * and † indicates *p* < 0.05.

We performed canonical pathway analysis of the top 8,000 DEGs between the HSR- and LSR-Cont. In contrast to SZ group, DEGs involved in DNA damage repair were not enriched in control. We compared seven molecules that significantly differed between HSR- and LSR-SZ for 4 groups: HSR- and LSR-SZ, and HSR- and LSR-Cont. *MRE11*, *BRCA2*, *ATRX*, *POLA1*, *LIG1,* and *GEN1* were significantly increased in the HSR-Cont compared to those in the LSR-Cont, but only *RPA1* was significantly decreased in the HSR-Cont (Figure 5). This result suggests that the relationship observed in the SZ cases also existed equally in controls. Furthermore, the mRNA expression levels of MRE11, BRCA2, ATRX, LIG1, and GEN1 were significantly higher in the HSR-SZ than in the HSR-Cont, and RPA1 expression tended to be lower in the HSR-SZ than in the HSR-Cont. In contrast, there were no significant differences in the seven molecules between the LSR-SZ and -Cont groups.

## 4. Discussion

In this study, we performed a hierarchical cluster analysis of SZ based on gene expression patterns of stress-responsive molecules in the postmortem brain and then successfully divided the patients with SZ into HSR- and LSR-SZ. We also found that the family history of SZ tended to be lower in the HSR-SZ. Canonical pathway analysis of DEGs between these groups showed the striking findings that they were most highly associated with the DSB repair pathway, and mRNA expression levels of individual factors in this pathway generally tended to be higher in the HSR-SZ. Moreover, we detected DNA break ends by TUNEL staining of brain tissue sections, suggesting that there may be less stainable DNA damage in HSR-SZ than in LSR-SZ. In the present study, we revealed for the first time that DEGs between the HSR- and LSR-SZ were most enriched in the DSB repair pathway.

The LSR-SZ contained significantly more cases of family members with SZ than the HSR-SZ. This supports our hypothesis that genetic influences on susceptibility to SZ are greater in the LSR-SZ than in the HSR-SZ. Compared to familial SZ, it is considered that environmental factors may play more significant role in its pathogenesis in sporadic SZ (43). Our results, in which patients with SZ with a family history were more common in clusters that were thought to be less affected by stress as a representative of environmental factors, may support previous reports based on family history from a different perspective. Although comparisons of familial and sporadic SZ have previously reported more intense negative symptoms (44–46), younger age of onset (47, 48), and lower gray matter density in the thalamus (49) in familial SZ, this study found no differences in psychiatric symptoms and age of onset between the HSR- and LSR-SZ. Genetic variants associated with SZ have been reported to increase vulnerability to stress (50), and an LSR-SZ, which is thought to have a greater genetic influence on the pathology, may show the same degree of psychiatric symptoms as the HSR-SZ, even with minor stress. Additionally, childhood abuse and traumatic experiences have been reported to increase the risk of psychosis (51), and it is likely that the HSR-SZ was under severe stress and, thus, had no significant change in the age of onset from the LSR-SZ, which has more genetic factors. There were no significant differences in confounders, such as cause of death and PMI, between the HSR- and LSR-SZ, but RIN was significantly lower in the HSR-SZ. RIN is often used as an indicator of mRNA quality and has been reported to be affected by PMI and agonal state (52). It has been reported that mRNA expression varies with RIN (53), and our other study showed that the genes whose expression changes in correlation with RIN included genes involved in the immune system (54). Therefore, this result may have been reflected in the stress-responsive molecules used in the cluster analysis, as many of them were also related to inflammation.

IPA analysis of DEGs between the HSR- and LSR-SZ showed that DEGs were most accumulated in pathway of DSB repair by homologous recombination. We found that in the HSR-SZ, gene expression of molecules involved in DSB repair by homologous recombination was significantly higher in six out of seven molecules than in the LSR-SZ, suggesting that DNA damage and repair were enhanced in the HSR-SZ. DNA damage and repair in the brain and its pathogenicity have attracted considerable attention recently. It has been noted that the human brain, especially neurons, is susceptible to physiological DNA damage, which occurs frequently during the generation of long transcripts (55). Furthermore, mature neurons, which are non-dividing, do not undergo proliferation-dependent DNA repair and removal of damaged cells and therefore lead to the accumulation of DNA damage such as DNA single-strand breaks and DSB (55, 56). It has also been reported that in mice learning by exploration of a novel environment or fear conditioning causes DSBs in both neurons (57) and glial cells (58). Stott et al. demonstrated that repeated cycles of DSBs and their repair might increase *de novo* gene mutations in neurons and glia, potentially increasing the risk of neurological and psychiatric disorders (58). Furthermore, DSBs and single-strand breaks are thought to affect DNA methylation and demethylation and may play pivotal roles in the development of SZ (55, 59). Thus, based on findings regarding the role of DNA damage and repair in pathogenesis, it is considered that physiological DNA breaks, increased neural activity mediated by glutamate receptors (60), and oxidative stress are the causes of DNA damage in the brain. Since the HSR-SZ is thought to be more affected by stress, oxidative stress and increased neural activity due to psychosocial stress likely have a greater effect and cause DSBs, resulting in the activation of DSB repair. In addition, homologous recombination repair is less likely to occur in neurons, which are non-dividing cells (61), and this result mainly reflects the changes in glial cells.

**Figure 6:**
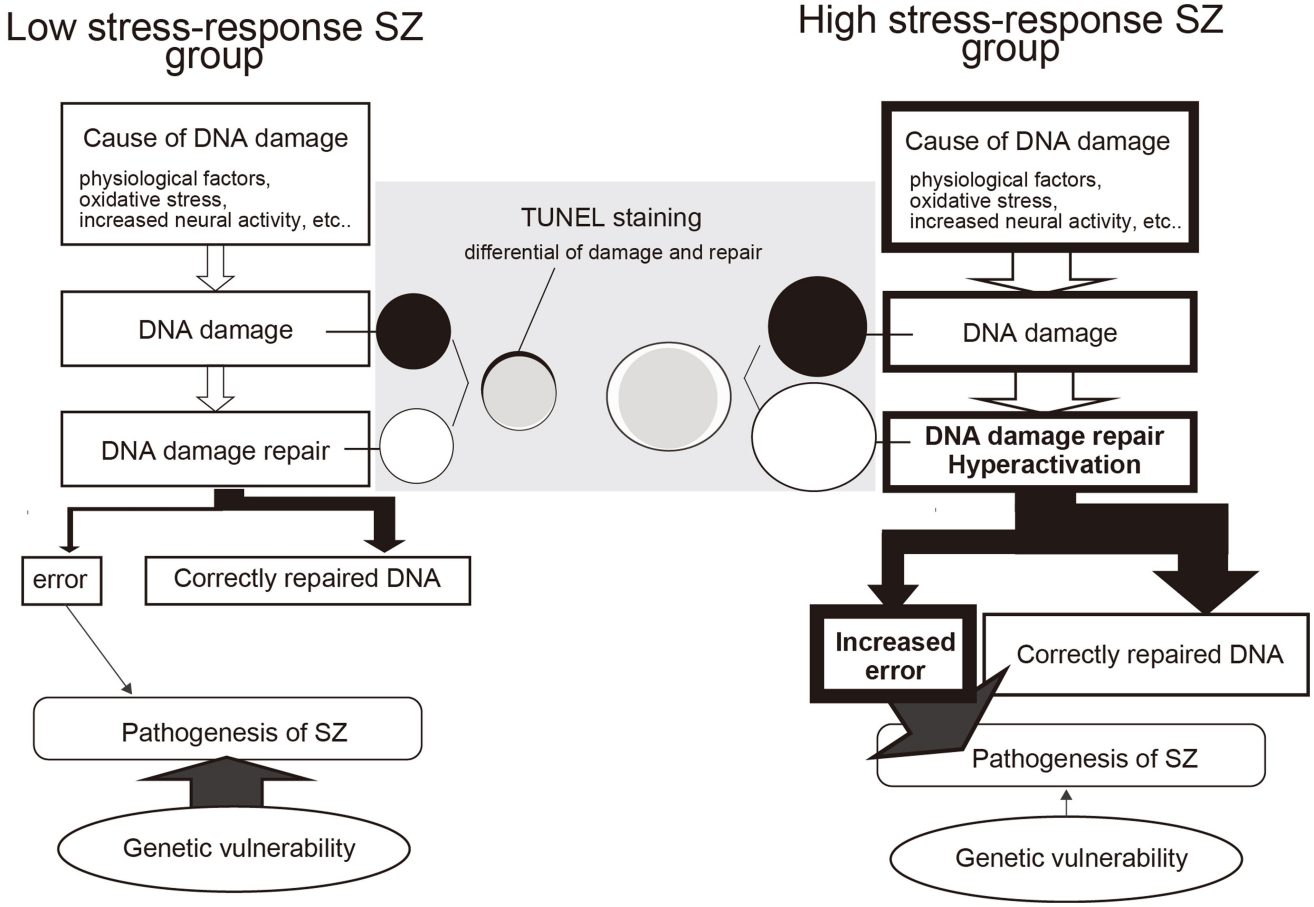
Model of the difference between DNA damage repair and the influence of genetic factors in high and low stress-response schizophrenia (SZ) groups. In the high stress-response SZ group, the effects of psychosocial stress are more significant and may increase DNA damage. This induces hyperactivity of DNA repair mechanisms, resulting in frequent cycles of DNA damage and repair. As a result, errors such as *de novo* mutations and methylation abnormalities increase, contributing to the development of SZ. In contrast, the low stress-response SZ group does not experience increased stress-induced DNA damage and therefore does not increase errors as a coproduct of DNA damage repair. However, they do have a genetic vulnerability, which may lead to the development of SZ even in the absence of severe stress. TUNEL staining is thought to detect differences in DNA damage (black circles) and repair (white circles). In the low stress-response group, detectable black areas of DNA damage remain, whereas, in the high stress-response group, no TUNEL-detectable DNA damage is likely due to the hyperactive state of DNA repair mechanisms.

Although the mRNA expression of molecules involved in DSB repair was upregulated in the HSR-SZ, suggesting that DSBs are increased, we observed two cases where the percentage of TUNEL-stained positive cells was higher only in the LSR-SZ. Since DNA damage and repair are thought to occur regularly in neurons and glial cells, it can be assumed that positive TUNEL staining reflects the differential between damage and repair. Therefore, repair mechanisms may be maintained at high levels in HSR-SZ to cope with repeated DNA damage, resulting in fewer cells that can be detected as positive by TUNEL staining as the damaged DNA was over-repaired and undetectable. Based on these results, we hypothesize that in the HSR-SZ, the excessive repetition of DNA damage and repair increases *de novo* mutations and epigenomic changes, such as DNA methylation, leading to abnormalities in key molecules of SZ pathogenesis and its development (Figure 6). In contrast, the LSR-SZ, in this hypothesis, has an innate vulnerability to these key molecules; hence, it can be considered that even stresses that are not as severe as those in the HSR-SZ can lead to disease onset (Figure 6). Whether somatic mutations and epigenomic modifications are truly increased in the HSR-SZ compared to that in the LSR-SZ or controls is unclear and requires further investigation. In addition, TUNEL-positive cells detected in both groups were glial cells, again suggesting that DNA damage and the repair cycle may be specifically enhanced in glial cells. In mice, a robust transcriptional response to glucocorticoids was induced in astrocytes, microglia, and oligodendrocytes but not in neurons, and some of these genes showed a high DSB marker (58). The GR mRNA expression level was used as stress-responsive molecules in this study, which may reflect the effects of glucocorticoids on glial cells. However, due to the small sample size, the results of TUNEL staining were preliminary and need to be validated on a larger sample.

Similar to the approach taken for SZ, we classified the control group into HSR- and LSR-Cont based on the expression levels of stress-responsive molecules. The seven molecules involved in DSB repair by homologous recombination that showed differences between the SZ groups were also significantly different between healthy groups. Furthermore, the molecules that were up- and downregulated in the more stressed group were consistent with those in both the SZ and control groups. This suggests that stress-induced enhancement of the DSB repair pathway occurs in healthy controls *via* a mechanism similar to that in SZ. However, when the above expression changes were compared between the HSR-SZ and -Cont, they were more remarkable in the HSR-SZ. These results indicate that the stress experienced by the HSR-SZ may have been more severe than that experienced by the HSR-Cont.

This study has some limitations. First, our study population was relatively small, and our findings require verification using larger samples. Next, since this study used postmortem brains, it is possible that the study may have been influenced by agonal states, physical illness, medications, including antipsychotics, and the long-term effects of SZ itself. As the patients whose brains were used in this study had died during the usual clinical course, cortisol levels and stress rating scales for when they were alive were unavailable, and we could not classify the cases by these data. Although our study did not find significant differences in demographic data, such as cause of death or PMI, except RIN, between the HSR- and LSR-SZ, there may be other confounding factors involved in stress and DNA damage and repair. In this study, we examined the differences in CPZ-eq of total dosage of antipsychotic medications between the two SZ groups, but not by their type. We also did not have information on the history of anti-inflammatory medications. Therefore, further studies examining the effects of these medications are needed. Furthermore, because this was a postmortem brain study, it is unclear if the enhanced DSB repair pathway occurred prior to the onset of SZ. It is possible that the DSB repair mechanism may be enhanced in some patients with SZ as a result of pathology. Additionally, we did not have paraffin sections of control brains; so we could not perform TUNEL staining on them and compare them to SZ. Moreover, since the analyzes in this study were based on mRNA expression levels only, its biological significance is minor if there is no correlation with protein expression. Thus, the analyzes based on protein expression levels would be necessary in future studies.

In conclusion, much remains unknown about the molecular neuroscientific mechanisms by which environmental factors, such as psychosocial stress, influence the onset and progression of SZ. However, the present study provides evidence for the existence of a subgroup within the SZ in which the DNA damage and repair cycle may be hyperactivated under the influence of stress. Our results may provide insights into novel strategies for the biological treatment of environmentally induced SZ.

## Data availability statement

The datasets presented in this article are not readily available because of privacy restrictions. Requests to access the datasets should be directed to the corresponding author/s.

## Ethics statement

The studies involving human participants were reviewed and approved by Ethics Committee of Fukushima Medical University and Ethics Committee of Niigata University. Written informed consent to participate in this study was provided by the participants’ legal guardian/next of kin.

## Author contributions

RS, MH, RI, AN, HT, and YK designed this study. RS, MH, and RI performed experiments. RS, RI, AN, MH, HH, AK, HY, and YK collected the postmortem brain samples and clinical information. RS, RI, MH, and YK performed statistical analyzes. RS wrote the first draft. All authors contributed to and approved the final manuscript.

## Funding

This work was supported by Japan Agency for Medical Research and Development (AMED)–JP22dm0207074 (YK), AMED–JP22wm0425019(AK), AMED–JP22wm0425019 (HY); the Grants-in-Aid for Scientific Research (C) from the Ministry of Education, Culture, Sports, Science, and Technology of Japan under Grant Number JP19K08053 (YK); the Grant-in-Aid for Scientific Research on Innovative Areas from the Ministry of Education, Culture, Sports, Science, and Technology of Japan under Grant Number JP21H00180 (YK), This study was also supported by the Collaborative Research Project of the Brain Research Institute, Niigata University under Grant Number 22002 (YK).

## Conflict of interest

The authors declare that the research was conducted in the absence of any commercial or financial relationships that could be construed as a potential conflict of interest.

## Publisher’s note

All claims expressed in this article are solely those of the authors and do not necessarily represent those of their affiliated organizations, or those of the publisher, the editors and the reviewers. Any product that may be evaluated in this article, or claim that may be made by its manufacturer, is not guaranteed or endorsed by the publisher.

## Footnotes

^1^http://www.usadellab.org/cms/index.php?page=trimmomatic

## Supplementary material

SUPPLEMENTARY FIGURE S1 Box-and-whisker plot of gene expression levels of stress-responsive molecules of two Cont subgroups showing the median (thick line in box), 25th and 75th percentiles (box ends), and minimum and maximum (whiskers) values. HSR-Cont, high stress-response Cont group; LSR-Cont, low stress-response Cont group; CPM, count per million; * indicates *p* < 0.05.

SUPPLEMENTARY TABLE S3 The mRNA expressions of stress-responsive molecules of SZ and Cont. CPM, count per million; SZ, schizophrenia; Cont, control; IL, interleukin; TNF, tumor necrosis factor; GR, glucocorticoid receptor; BDNF, brain-derived neurotrophic factor; SYP, synaptophysin; IFN, interferon; S100B, S100 calcium-binding protein B; SOD, superoxide dismutase; Apo, apolipoprotein; PSD, postsynaptic density protein; SNCA, α-synuclein; SNCB, β-synuclein; SNCG, γ-synuclein; SLC6A4, solute carrier family 6 member 4.

## Abbreviations

CPZ-eq: chlorpromazine equivalent of total antipsychotics
DIBS: diagnostic instrument for brain studies
DSB: DNA double-strand break
PMI: postmortem interval
RIN: RNA integrity number
TUNEL: Terminal deoxynucleotidyl transferase-mediated dUTP nick end labeling
