# Supplementary figures and images for: Evidence for increased DNA damage repair in the postmortem brain of the high stress-response group of schizophrenia
Source: Front Psychiatry. 2023 Aug 22;14:1183696. doi: 10.3389/fpsyt.2023.1183696 (PMC10478254; doi:10.3389/fpsyt.2023.1183696)

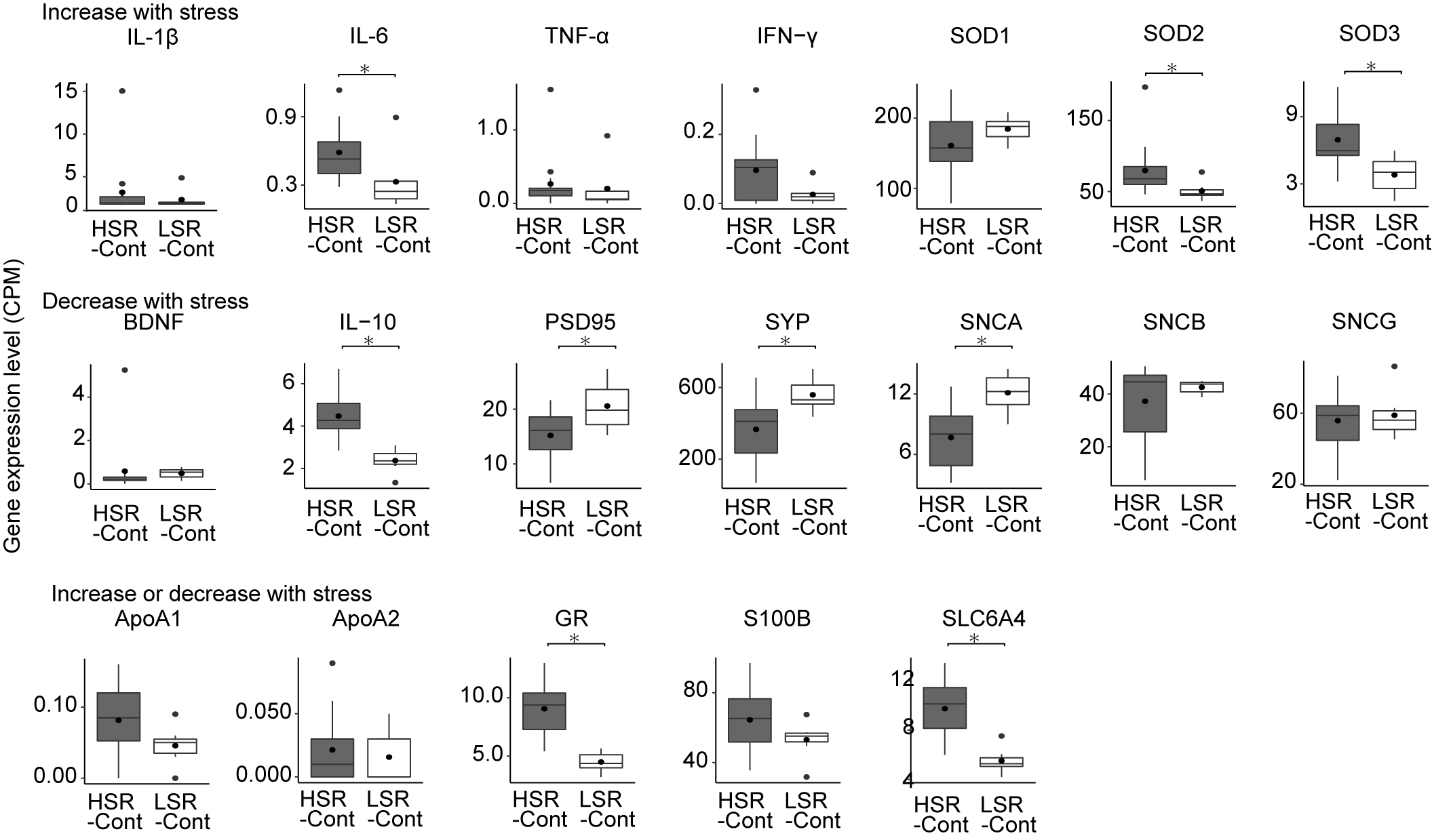

Supplement: SUPPLEMENTARY FIGURE S1 — Box-and-whisker plot of gene expression levels of stress-responsive molecules of two Cont subgroups showing the median (thick line in box), 25th and 75th percentiles (box ends), and minimum and maximum (whiskers) values. HSR-Cont, high stress-response Cont group; LSR-Cont, low stress-response Cont group; CPM, count per million; * indicates p < 0.05. [file Image_1.JPEG]
